# Bilateral Pyosalpinx Causing Obstructive Hydronephrosis: A Multimodality Imaging Approach with the Emphasis on the Diffusion-weighted Magnetic Resonance Imaging

**DOI:** 10.7759/cureus.6198

**Published:** 2019-11-19

**Authors:** Urban Čizmarević, Maja Podkrajšek, Nina Hanžič, Mitja Rupreht

**Affiliations:** 1 Radiology, University Medical Centre Maribor, Maribor, SVN

**Keywords:** pelvic inflammatory disease, pyosalpinx, hydronephrosis, diffusion weighted imaging, diffusion weighted magnetic resonance imaging, apparent diffusion coefficient

## Abstract

A 40-year-old female, who was being treated for a urinary tract infection, was admitted to the hospital due to a gradually increasing left flank colic pain. An ultrasound investigation detected right-sided hydronephrosis, and a computed tomography scan additionally showed large cystic changes in both the fallopian tubes, which were compressing the ureters and thus causing hydronephrosis. Subsequently, magnetic resonance imaging was performed, which demonstrated fluid-fluid levels inside the cystic changes. The differential diagnosis included a deep pelvic endometriotic cyst and a pyosalpinx.

## Introduction

Hydronephrosis refers to a dilatation of the pyelocaliceal system due to the problems with the outflow of the urine. Literally, it means "water inside the kidney" [1]. The cause for hydronephrosis can be functional (e.g., due to neurogenic bladder), but in most cases it is caused by an obstruction of the urine outflow. Hydronephrosis alone is typically asymptomatic and with a gradual onset. In the acute setting, the pain is usually present and the search for a kidney stone or an infection focus should be conducted [2]. Early diagnosis is important to avoid permanent damage to the kidneys.

## Case presentation

**Figure 1:**
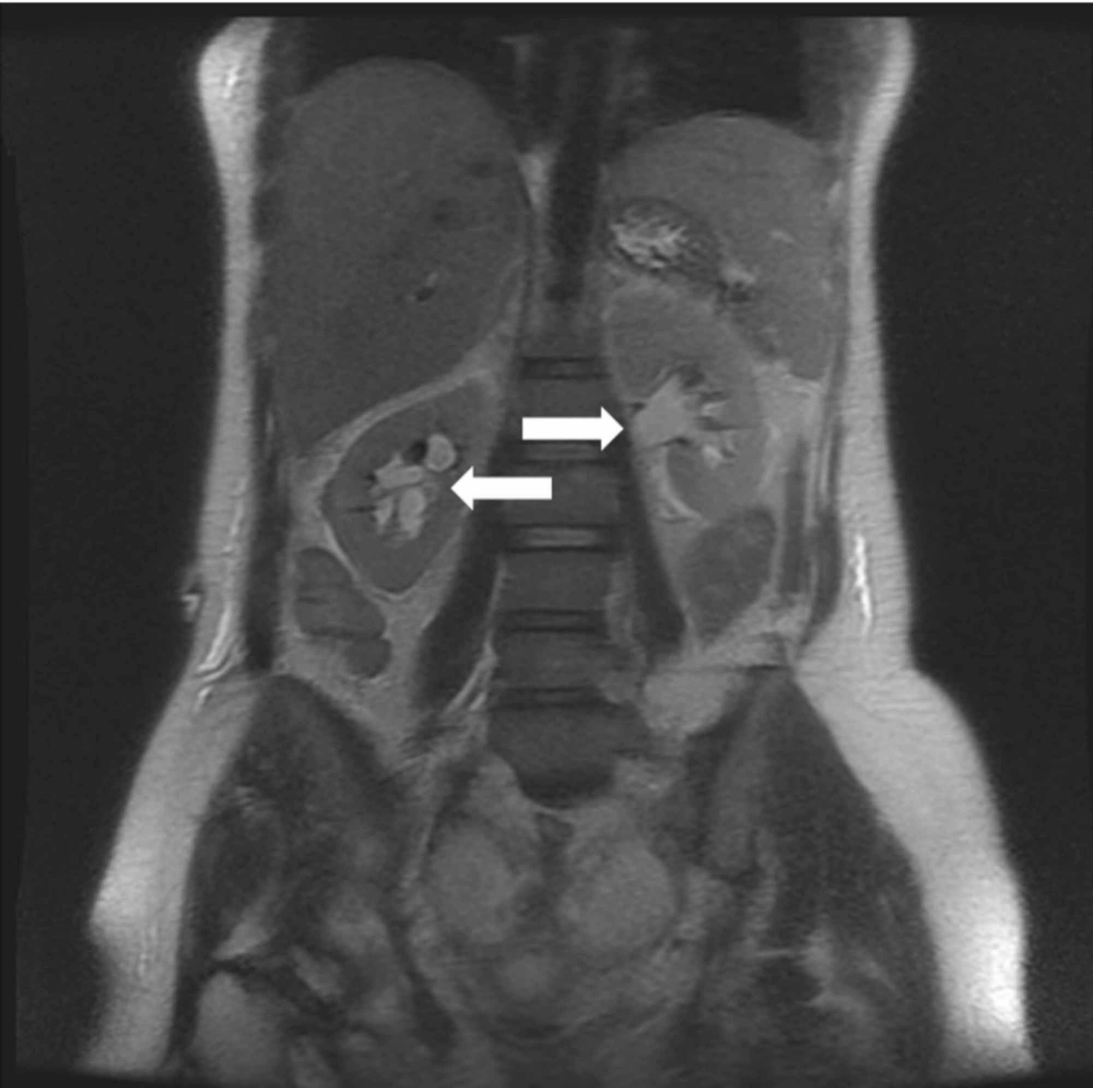
Coronal T2-weighted MRI of the abdomen demonstrating dilatation of the collecting systems (arrows), confirming hydronephrosis. MRI, magnetic resonance imaging

**Figure 2:**
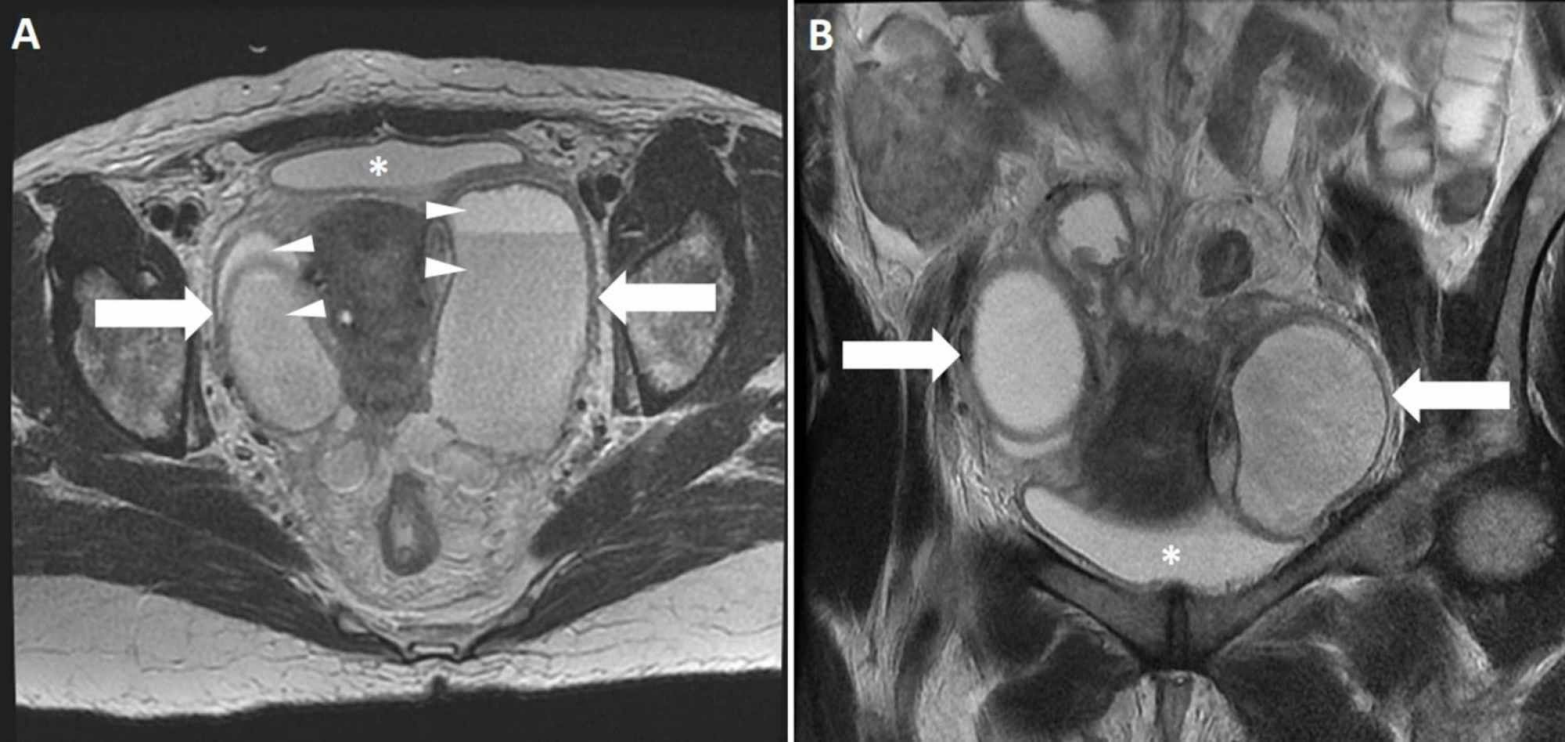
Axial (A) and coronal (B) T2-weighted MRI of the pelvis demonstrating bilateral adnexal cystic changes (arrows). Fluid-fluid levels (arrowheads) are seen in the axial view (A). The signal intensity (SI) of the fluid signal is similar to the SI of the fluid inside the bladder (asterisk). MRI, magnetic resonance imaging

A 40-year-old female with a five-year history of uterine fibroids experienced left-sided flank pain radiating towards the inguinal region. She had no dysuria or hematuria, but she did notice increased urinary frequency. Because the problems started during the weekend, she decided to start with self-administration of ciprofloxacin, before seeing her family doctor on Monday. The blood tests showed leukocytosis (17 x 10^9^/L) and increased values of C-reactive protein (CRP) (210 mg/L). Her physician additionally prescribed co-amoxiclav, but since the pain was getting gradually worse, she was admitted to the hospital in the evening. Despite the high inflammatory markers, she had no fever. Her general physical examination was unremarkable, aside from some tenderness at the right costovertebral angle (CVA). She was given an intravenous (IV) infusion of metamizole and ketoprofen. A gynecological examination revealed an enlarged and tender uterus. Due to the pain, adnexal palpation was not performed, but tenderness was assumed. A bedside ultrasound (US) investigation detected hydronephrosis, stage 1 or 2 on the right side, as well as a possible small (5-6 mm) stone at the distal end of the right ureter. A non-contrast computed tomography (CT) scan was performed for further investigation. It did not confirm the presence of the stone but it showed bilateral adnexal cystic changes, with a compression of the right ureter causing hydronephrosis. Therefore, a percutaneous nephrostomy (PCN) catheter was inserted in the collecting system of the right kidney. For further evaluation of the cystic changes, a magnetic resonance imaging (MRI) scan was performed, which showed a clear dilatation of the collecting system in both kidneys (Figure 1), as well as fluid-fluid levels inside the adnexal cystic changes bilaterally (Figure 2).

**Figure 3:**
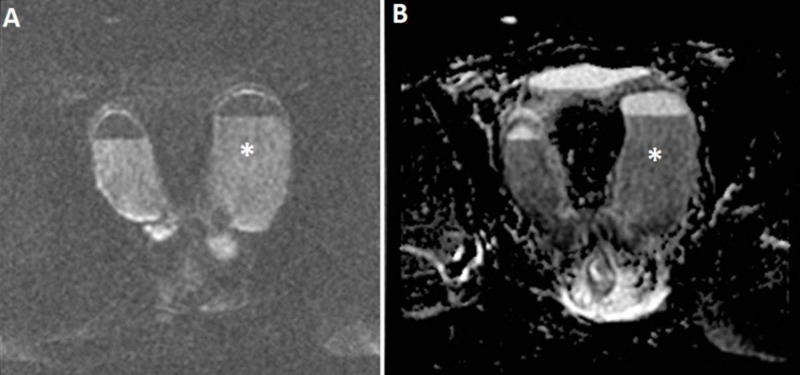
Coronal DW-MRI at the b-value of 800 s/mm2​​​​​​​ (A) and ADC map (B) of the cystic changes. The bottom level (asterisk) shows a restriction of diffusion. ADC, apparent diffusion coefficient; DW-MRI, diffusion-weighted magnetic resonance imaging

Additional diffusion-weighted imaging (DWI) sequence was performed with b-values of 0 and 800 s/mm^2^ (Figure 3). It again demonstrated the fluid-fluid levels and revealed that the bottom fluid level has a lower signal intensity. The difference was even more pronounced on the apparent diffusion coefficient image with a lower signal indicating restricted diffusion (Figure 3B) owing to a higher amount of cellular parts. The differential diagnosis consisted of a deep pelvic endometriotic hemorrhagic cyst and a pyosalpinx, but based on the signal intensity of the fluid at the DW-MRI, a pyosalpinx was more probable.

The MRI also revealed signs of small bowel ileus, and therefore laparoscopic surgery with a right-sided adnexectomy, left-sided pyosalpinx drainage, and abdominal adhesiolysis was performed. Two days after the surgery, high inflammatory parameters (leukocytes 18 x 10^9^/L, CRP 188 mg/L, procalcitonin 17 ng/mL) were still present, and treatment with ceftriaxone and metronidazole was replaced with meropenem, which resulted in normalization of the parameters.

## Discussion

When hydronephrosis is associated with an infection (pyonephros), the expected signs and symptoms include flank pain, fever, CVA tenderness, and urinary symptoms (pyuria, dysuria, increased frequency). The loin pain due to stretching of the renal pelvis in acute hydronephrosis is called Dietl's crisis [3]. The most common cause of obstruction in adults is a kidney stone. Besides intrinsic obstructions, any compression of the urinary tract structures causes increased pressure in the renal system. Benign prostatic hyperplasia and several malignant tumors and metastases are the leading causes; however, other more rare causes such as a pyosalpinx are also possible [3,4]. As the increased pressure acts on the sensitive tissues of the filtration system and impedes the normal urine outflow, it can lead to an infection, the formation of stones, or, eventually, damage to the tissue [3]. CT without contrast is usually the initial imaging modality for suspected hydronephrosis since it has excellent sensitivity (97%) and specificity (96%) for the detection of urolithiasis [5]. If the patient's kidney function allows the use of IV contrast, then CT urography can detect renal collecting system anatomy and patency abnormalities, differentiate between partial and complete obstructions, as well as evaluate the urothelial thickness [4]. When suspecting a cause associated with the soft tissues, MRI is the best option. If the underlying cause of acute obstructions of the upper urinary tract can not be resolved, typically the PCN procedure is performed to preserve kidney function. The success rate for PCN is very high, at 96%-99% [6,7]. The treatment for chronic malignant obstructions is usually an insertion of a ureteric stent [8].

The probable course of events in the presented case has been a pelvic inflammatory disease (PID) progressing to a formation of pyosalpinx, which pressed on the ureters causing hydronephrosis. PID is an infection of the female upper genital tract, which mostly affects sexually active women. In 75%-90% of cases, PID is caused by sexually transmitted pathogens, most commonly by *Neisseria gonorrhoeae *or *Chlamydia trachomatis* [9]. The rest of the cases are caused when enteric or respiratory pathogens colonize the genital tract [10]. The symptoms of PID usually occur during or shortly after the period and include lower abdominal or pelvic pain, abnormal vaginal discharge, intermenstrual or post-coital bleeding, dyspareunia, and dysuria [11]. Fever, as well as other systemic signs, is usually not present [10], as was the case in our patient. Asymptomatic subclinical infections are also common and a frequent cause of infertility [12-14]. The inflammatory damage caused by the infection can lead to scarring and adhesions and may cause obstruction of the fallopian tubes [10]. Pyosalpinx and a tubo-ovarian abscess may then form. PID is usually bilateral [15], which can explain the almost symmetrical formation of the pyosalpinxes in our patient, although in the study by Kim et al. 67% of patients with PID who developed a pyosalpinx had unilateral pyosalpinx formation only [16]. As in the presented case, the diagnosis of pyosalpinx is usually established by a multimodality imaging approach. First, the US detected a urinary tract obstruction with hydronephrosis. CT excluded the stone as the cause of the obstruction but pointed in the direction towards adnexal masses. These could be further evaluated with the IV administration of iodine contrast but at the expense of ionizing radiation and with the risk of further damage to the renal parenchyma. Therefore, in the presented case, the key examination leading to the correct diagnosis was DW-MRI, which provides a non-invasive imaging technique to assess the random diffusion of water molecules. Owing to the different diffusion properties of the blood and pus, differentiation between them is possible with the DWI [17]. In the presented case, the diagnosis made with DW-MRI was then also confirmed laparoscopically. Due to the possibility of severe sequelae, PID treatment threshold should be low and include broad-spectrum empirical antibiotic coverage. Although there is no gold standard therapy, most guidelines suggest cefoxitin and doxycycline as the starting regiments [18]. If there are complications, surgery might be needed.

## Conclusions

In sexually active females with urinary tract infection and obstruction, the rarer pathology, such as the pyosalpinx, has to be taken into account. In such cases, a multimodality imaging approach is necessary with advanced MRI techniques, such as DW-MRI, leading to proper diagnosis and treatment.
